# Anti-Inflammatory Effects of (3*S*)-Vestitol on Peritoneal Macrophages

**DOI:** 10.3390/ph15050553

**Published:** 2022-04-29

**Authors:** Bruno Bueno-Silva, Manuela Rocha Bueno, Dione Kawamoto, Renato C. Casarin, João Marcos Spessoto Pingueiro, Severino Matias Alencar, Pedro Luiz Rosalen, Marcia Pinto Alves Mayer

**Affiliations:** 1 Department of Microbiology, Institute of Biomedical Sciences, University of São Paulo, São Paulo 05508-900, SP, Brazil; manirocha@gmail.com (M.R.B.); dionek@usp.br (D.K.); mpamayer@icb.usp.br (M.P.A.M.); 2 Dental Research Division, Guarulhos University, Guarulhos 07023-070, SP, Brazil; spessoto.jm@gmail.com; 3 Piracicaba Dental School, University of Campinas-UNICAMP, Piracicaba 13414-903, SP, Brazil; casarinrcv@yahoo.com.br (R.C.C.); pedro@unicamp.br (P.L.R.); 4 College of Agriculture “Luiz de Queiroz” (ESALQ/USP), University of São Paulo, Piracicaba 13418-900, SP, Brazil; smalencar@usp.br

**Keywords:** inflammation, natural products, propolis

## Abstract

The isoflavone (3*S*)-vestitol, obtained from red propolis, has exhibited anti-inflammatory, antimicrobial, and anti-caries activity; however, few manuscripts deal with its anti-inflammatory mechanisms in macrophages. The objective is to elucidate the anti-inflammatory mechanisms of (3*S*)-vestitol on those cells. Peritoneal macrophages of C57BL6 mice, stimulated with lipopolysaccharide, were treated with 0.37 to 0.59 µM of (3*S*)-vestitol for 48 h. Then, nitric oxide (NO) quantities, macrophages viability, the release of 20 cytokines and the transcription of several genes related to cytokine production and inflammatory response were evaluated. The Tukey–Kramer variance analysis test statistically analyzed the data. (3*S*)-vestitol 0.55 µM (V55) lowered NO release by 60% without altering cell viability and diminished IL-1β, IL-1α, G-CSF, IL-10 and GM-CSF levels. V55 reduced expression of *Icam-1*, *Wnt5a* and *Mmp7* (associated to inflammation and tissue destruction in periodontitis) and *Scd1*, *Scd2*, *Egf1* (correlated to atherosclerosis). V55 increased expression of *Socs3* and *Dab2* genes (inhibitors of cytokine signaling and NF-κB pathway), *Apoe* (associated to atherosclerosis control), *Igf1* (encoder a protein with analogous effects to insulin) and *Fgf10* (fibroblasts growth factor). (3*S*)-vestitol anti-inflammatory mechanisms involve cytokines and NF-κB pathway inhibition. Moreover, (3*S*)-vestitol may be a candidate for future in vivo investigations about the treatment/prevention of persistent inflammatory diseases such as atherosclerosis and periodontitis.

## 1. Introduction

Inflammation is activated to protect the human body from damages and/or pathogens; however, when unrestrained, it triggers extensive neutrophil enrollment and massive macrophage stimulation, causing autoimmune cell death and severe immune pathologies [1]. Despite it being part of the normal body response to eliminate pathogens and promote tissue regeneration, the poorly regulated inflammatory response is associated with several diseases, as well as periodontitis, cardiovascular disorders and diabetes, among others.

Chronic inflammation takes place for a long time (weeks to months) and is correlated with the presence of macrophages and lymphocytes, vascular creation, fibrosis and tissue damage. For the duration of inflammation, immune cells, particularly macrophages, can be activated in a variety of ways. One of them is the antigen (s) detection of attacking bacteria, such as lipopolysaccharide (LPS). The recognition takes place by toll-like receptors (TLR), causing stimulation of nuclear factor-kappa B (NF-κB) and mitogen-activated protein kinases (MAPk) intracellular-signaling pathways. In sequence, both NF-κB and MAPk pathways activate associated genes related to the inflammation, such as iNOS and the up-regulation of pro-inflammatory and the suppression of anti-inflammatory genes [2].

Recognizing the importance of restoring tissue homeostasis, the search for new compounds that can modulate the host’s immunoinflammatory response arises, focusing on controlling/resolving inflammation [3,4,5,6,7,8,9,10,11,12], especially with an emphasis on the natural products as possible sources for the isolation of bioactive compounds with pharmacological actions [13,14].

Brazil holds diverse vegetation along its vast extension. As a result, numerous natural products with distinct pharmaceutical properties may be discovered [13,15]. Among them, several different propolis can be found there. Propolis may vary its chemical composition and biological properties according to its geographical location [16]. For example, Brazilian green propolis is found in Minas Gerais state (region southeast) [16], and Brazilian organic propolis is obtained in Parana and Santa Catarina states (south region) [17]. In 2008, our research group published the botanical origin of Brazilian red propolis, classifying it as the 13th type of propolis found in Brazilian territory, precisely, in Alagoas state, northeast of Brazil [15]. Thus, the literature presents several manuscripts that isolated several compounds with distinct pharmacological properties [12,18,19,20]. Among them, (3*S*)-vestitol stands out.

Among the several compounds isolated from natural products studied over the literature, (3*S*)-vestitol is an isoflavone, present in Cuban and Brazilian red propolis [15,18,19,20,21,22] and in Licorice root preparations, that are frequently used as dietary supplements by women of menopausal age instead of additional chemical hormone treatment, and also in *Dalbergia ecastaphyllum* (L.) *Taub* (Engler and Plantl), a Leguminosae plant [23]. This molecule already presented anti-inflammatory activity over neutrophils, antibacterial and anti caries properties [15,19,20,22]. A recent manuscript from our research group demonstrated that (3*S*)-vestitol acts on Raw 264.7 macrophages by impeding the NF-κB pathway activation and decreasing pro-inflammatory cytokines [20]. However, its effects and mechanisms over peritoneal macrophages collected from mice are still unclear.

Therefore, the present work aimed to assess the (3*S*)-vestitol anti-inflammatory activity on LPS-activated peritoneal macrophages and elucidate its molecular mechanisms of anti-inflammatory activity.

## 2. Results

### 2.1. NO Quantification and Cell Viability

**Figure 1:**
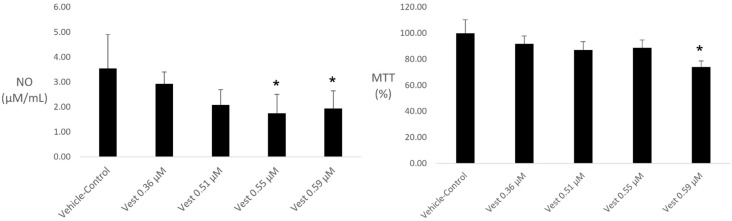
(3*S*)-vestitol effects on NO release and macrophage viability of LPS-stimulated peritoneal cells (n = 6). * statistical difference compared to control group by one-way ANOVA plus Tukey post-hoc test, *p* <0.05).

(3*S*)-vestitol 0.59 µM was the unique concentration promoting reduction on cell viability, although lower concentrations (0.36 and 0.51 µM) were not able to reduce NO levels. Thus, (3*S*)-vestitol 0.55 μM (V55) was shown to be the lowest concentration capable of reducing NO production without interfering with macrophages viability (Figure 1).

### 2.2. Cytokines Production

**Figure 2:**
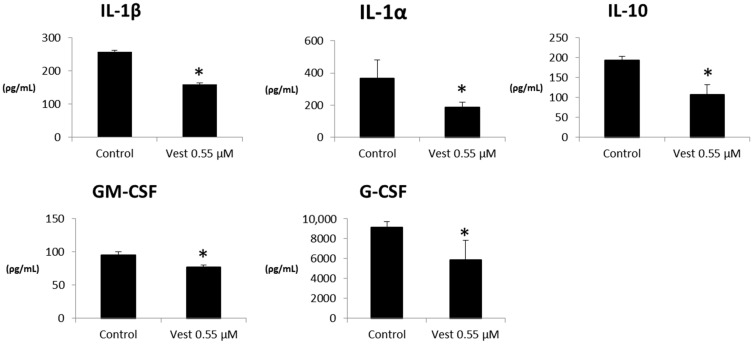
Cytokines profile of LPS-activated peritoneal macrophages treated with (3*S*)-Vestitol 0.55 µM (n = 6). * statistical difference compared to vehicle-control group (Mann-Whitney, *p* <0.05).

V55 was able to decrease the inflammatory cytokines IL-1β, IL-1α, GM-CSF, G-CSF; however, it also decreased the amounts of anti-inflammatory IL-10 (*p* < 0.05), see Figure 2. The other tested cytokines did not present statistical significance (data not shown).

### 2.3. Gene Expression

**Figure 3:**
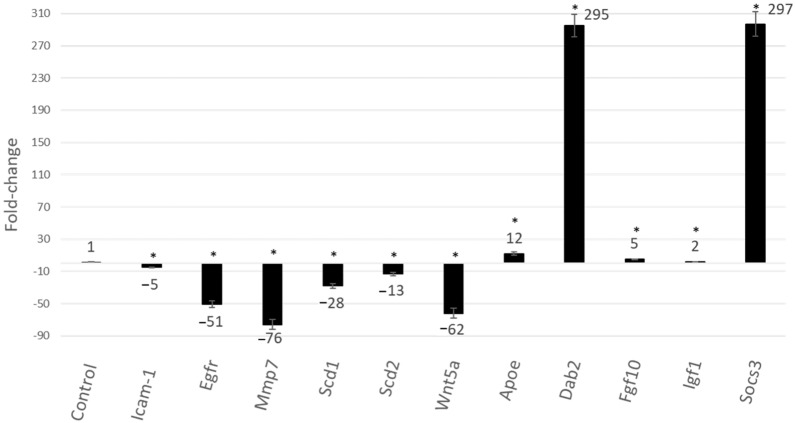
Gene expression analysis of LPS-activated peritoneal macrophage treated with (3*S*)-vestitol 0.55 µM. Statistical Analysis: Student’s t-test was conducted to assess statistical significance between vehicle-control and V55 group utilizing mean CT values obtained from the triplicate samples (*p* < 0.05). * statistical difference compared to control group by statistical analysis-SABiosciences Technical Core (SABiosciences/Qiagen Corp., Frederick, MD, USA).

V55 diminished the expression of *Icam-1*, Egfr, *Wnt5a* and *Mmp7* (related to inflammation and tissue damage in periodontal disease) and *Scd1*, *Scd2*, *Egf1* (related to atherosclerosis). Moreover, V55 treatment enhanced *Socs3* and *Dab2* expression (inhibitors of cytokine signaling and NF-κB pathway), *Apoe* (associated to atherosclerosis control), *Igf1* (encoder a protein with analogous effects to insulin) and *Fgf10* (fibroblasts growth factor) (*p* < 0.05), see Figure 3.

## 3. Discussion

Inflammation is a host defensive reaction aiming to protect the human body against different stimuli such as mechanical, thermal and infective stimuli. However, when uncontrolled, it may harm the organism [24]. Therefore, molecules that modulate the inflammatory response are of great interest to industry and science. Herein, we demonstrate the immune-modulatory effects of (3*S*)-vestitol, an isoflavonoid obtained from Brazilian red propolis that has anti-inflammatory properties. (3*S*)-vestitol 0.55 µM was the unique tested concentration that decreased nitric oxide production without affecting macrophage viability. Because of that, this concentration was chosen for the subsequent analysis (cytokine and gene expression). It is interesting to observe that the same concentration was the most effective in reducing nitric oxide release of LPS-activated RAW 267.4 macrophages [20].

The main cytokine inhibited by (3*S*)-vestitol treatment was IL1, which holds a key role in inflammation [25]. Its inhibition was indicated as the primary mechanism of the anti-inflammatory property of BRP on a distinct cell lineage RAW macrophage [26] and at neutrophils [10]. IL1 shows two different types—IL1α and IL1β. Both IL1 subtypes tie to the same IL1RI receptor. Interleukin decreased-release is vital for the (3*S*)-vestitol anti-inflammatory action, leading to the inactivation of some transcription factors such as nitric oxide synthase (iNOS) and nuclear factor kappa B (NF-κB) [25,27]. Although we did not observe repression on the transcription of iNOS, the decreased release of IL1 may account for the decrease in nitric oxide production observed on (3*S*)-vestitol-treated macrophages.

Granulocyte colony-stimulating factor (G-CSF) and granulocyte-macrophage colony-stimulating factor (GM-CSF) are other cytokines analyzed. The inflammatory response to LPS promotes the macrophage GM-CSF-induced release of IL23, IL12, TNF and IL6, boosting the injuries of tissues. At the same time, IL1 increases G-CSF production to enroll neutrophils into the inflammatory focus. Furthermore, reducing GM-CSF positively impacts several disorders such as asthma, arthritis, psoriasis and inflammation of the lung [28,29]. In accordance with the literature, the same GM-CSF-reduced levels were observed as an effect of (3*S*)-vestitol treatment on RAW 264.7 macrophages [20].

As an unexpected result, (3*S*)-vestitol treatment provoked reduced levels of IL-10; however, a previous manuscript demonstrated the opposite, an increase of IL10 after (3*S*)-vestitol treatment [20]. This discordance between the present results and the literature may be due to the different cells that evaluated (3*S*)-vestitol effects. Here, we used a primary cell type obtained from mice.

**Figure 4:**
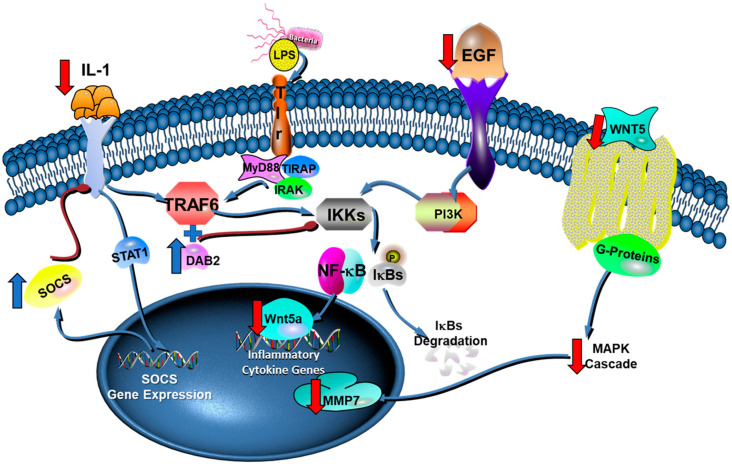
Molecular mechanisms of (3*S*)-vestitol anti-inflammatory actions in LPS stimulated peritoneal macrophages. “Red down arrow” indicates the decrease of genes transcription and/or pathway activation by (3*S*)-vestitol treatment while an “blue up arrow” indicated the up-regulation of gene expression and/or pathway activation due to (3*S*)-vestitol treatment. Adapted from Qiagen’s website [31].

The gene expression analysis (Figure 4) revealed that (3*S*)-vestitol treatment increased the expression of *Dab2*. The protein encoded by *Dab2* regulates the phenotypic switching in macrophages and inhibits NFkB translocation to the nucleus through DAB2 cooperating with TNF receptor-associated factor 6 (TRAF6) and reducing IκB kinase. Therefore, DAB2 is important for controlling inflammatory response intracellular signaling in the course of macrophages polarization. It is suggested that the management of DAB2 expression and function may have therapeutic potential for the therapy of acute and chronic inflammatory diseases [30].

SOCS3 is a suppressor of cytokine signaling. Thus, its increased expression also seems to be an outstanding finding since it turns off different cytokines receptors due to a process of negative feedback through a protein family called STATs [32]. Therefore, the release of cytokines may not be inhibited by (3*S*)-vestitol treatment, as observed in Figure 2; however, its pathway signaling may be hindered due to the increased expression of *Socs3*. In addition, its activation may also contribute to the downregulation of the NFkB pathway due to the deactivation of cytokines receptors that would lead to an increased expression NFkB pathway.

Moreover, an animal study demonstrated that SOCS-3-knockout mice had augmented alveolar bone loss after *P. gingivalis* infection. Once (3*S*)-vestitol up-regulated SOCS3 expression, it may be a relevant agent to reduce alveolar bone loss in an inflammatory condition such as arthritis and periodontal disease. A previous manuscript of our research group demonstrated the positive effects of (3*S*)-vestitol through different in vivo models [22]. (3*S*)-vestitol treatment reduced the transmigration of neutrophils to the inflammatory focus through reductions of rolling and adhesion of these cells to the wall of blood vessels. In line with the present manuscript, both studies found decreased expression of *Icam-1*, a protein related to the transmigration of leukocytes to the inflammation site.

Another interesting finding related to altered gene expression induced by (3*S*)-vestitol treatment was the inhibition of *Wnt5a* expression. This protein encoded by this gene is vital for the general inflammatory response of human macrophages in the course of sepsis. In addition, the expression of *Wnt5a* was dependent on toll-like receptor (TLR) intracellular signaling and on the NF-kB pathway in reaction to *Porphyromonas gingivalis* LPS stimulation of the human monocytic cell line THP-1 [33]. In addition, a recent manuscript demonstrated that *P. gingivalis* could potentially enhance inflammation through the capacity of its LPS to up-regulate the expression of *Wnt5a* [34]. Furthermore, the protein encoded by this gene suppresses osteoblastic differentiation of human periodontal ligament stem cell-like cells [35]. Therefore, these results suggest that the modulation of *Wnt5a* expression by *P. gingivalis* may perform an essential function in the periodontal inflammatory process. Therefore, since (3*S*)-vestitol decreased its expression, it might be helpful to control the inflammatory response in periodontal disease.

MMPs are the main proteins responsible for extracellular matrix remodeling and contribute to the pathogenesis of periodontitis through the destruction of periodontal tissue. Specifically, MMP7 was considered a new target in predicting poor wound healing in apical periodontitis [36] and, most recently, as novel salivary biomarkers for periodontitis [37]. Thus, decreased expression of *Mmp7* induced by (3*S*)-vestitol treatment could be considered another relevant finding that justifies future studies testing the effects of (3*S*)-vestitol on inflammatory diseases such as periodontitis.

Bacterial LPS stimulates the expression of the *Scd* gene to induce the formation of foam cells, macrophages containing large amounts of fatty acids that play an essential role in the development of atherosclerosis. This gene encodes an enzyme implicated in fatty acid biosynthesis, mainly oleic acid synthesis. Thus, SCD may be a key regulator of energy metabolism with a role in obesity and dyslipidemia. Four isoforms were identified in mice: SCD1 through SCD4. In contrast, only two isoforms, SCD1 and SCD5, have been found in humans. SCD1 is involved in insulin resistance, obesity and metabolic syndrome. Furthermore, its inhibition attenuated the accumulation of fatty acids and consequent liver injury and inflammation [36]. In addition, the protein encoded by the *Egr1* gene, whose expression is induced by LPS, performs a crucial function in the progress of atherosclerosis [38,39,40].

(3*S*)-vestitol treatment increased the expression of *Apoe*, *Igf1*, and *Fgf10*. *Apoe* encodes one protein with a vital role in preventing atherosclerosis. Its production was strongly positively regulated by TGF-β and repressed by bacterial lipopolysaccharide (LPS) and the inflammatory cytokines IFN-γ, TNF-α, and IL-1β [41]. Furthermore, it was shown that APOE inhibits the inflammatory responses of macrophages to TLR-4 and TLR-3 receptors through distinct mechanisms and that these inhibitory effects converged to the suppression of JNK and c-Jun activation, which are necessary for macrophage activation [42]. *Igf1* encodes a protein similar to insulin and *Fgf10*, a fibroblasts growth factor.

The (3*S*)-vestitol effect in reducing the expression of *Scd1*, *Scd2*, *Egr1* and its impact on increasing the *Apoe*, *Igf1*, and *Fgf10* expression demonstrates the positive effects that this molecule, derived from Brazilian red propolis, may have on metabolic diseases such as atherosclerosis and diabetes. Future studies should further evaluate these beneficial effects on the human body.

## 4. Materials and Methods

**Figure 5:**
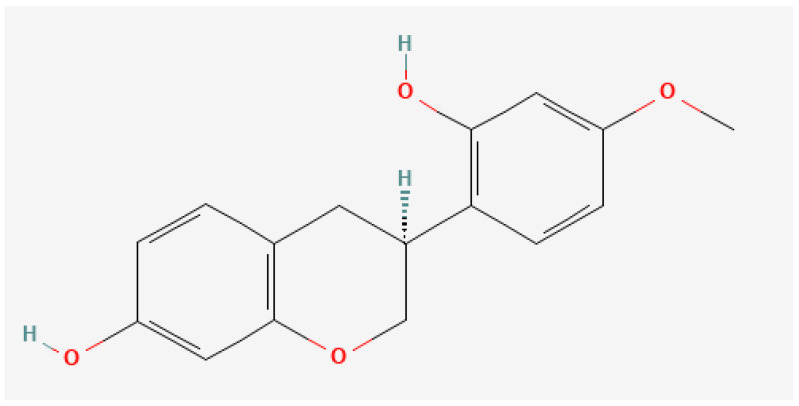
Chemical structure of (3*S*)-vestitol, isolated from Brazilian red propolis. (Figure obtained from [46]).

Samples of Brazilian Red Propolis (BRP) were harvested in the Maceió neighborhood (SL 09°39′57″, WL 35°44′07″), Alagoas state in the Brazilian northeastern area [18,43,44]. (3*S*)-vestitol (Figure 5) was obtained as earlier explained by Bueno-Silva et al. (2013) [18] and Oldoni et al. [45].

### 4.1. Growing of Eukaryotic Cell

Cells were acquired from the peritoneal cavity of mice C57BL6. Every test of the present manuscript followed the National Institutes of Health Guidelines for the Welfare of Experimental Animals and received authorization from the Institutional Committee for Ethics in Research with animals (protocol number: 176). The peritoneal cells were placed at 37 °C in a humidified environment with 5% CO_2_, for two hours and were cultured in bottles with culture medium RPMI-1640, complemented by 10% heat-inactivated fetal bovine serum (FBS); 10 mM Hepes, 0.05 mM b-mercaptoethanol, 1% Na-pyruvate, 11 mM sodium bicarbonate (NaHCO_3_), 1% glutamine and 1% penicillin/streptomycin. All these reagents were purchased from Sigma-Aldrich, USA. Then, the cells were sorted to pick macrophages utilizing a SuperMACS II Equipment Separator (Miltenyi Biotec, Bergisch Gladbach, Germany). After that, macrophage LPS stimulation was performed as follows.

### 4.2. Macrophages LPS-Activation in the Presence of (3S)-Vestitol

LPS (10µL) from *E. coli* serotype O111:B4 (Sigma, St. Louis, MI, USA) at 1 µg/mL were used to stimulate macrophages (2 × 10^5^ cells/well). At this moment, (3*S*)-vestitol-aliquots ranging from 0.37 to 0.59 µM (100–160 µg/mL) were added to the wells with macrophages. Then, the 96-well plate was placed during 48 h at 37 °C in 5% CO_2_. As a control, the DMSO-vehicle/LPS was used. Two independent experiments, each in triplicate, were performed [11].

### 4.3. Nitrite Oxid (NO) Production and CELL Viability

The contents of nitrite released in well supernatants were determined to verify NO release by cell culture through the Griess reagent method (Sigma, St. Louis, MI, USA), the results of which were expressed as mM of NO_2_. Cell viability was measured by 3-(4,5-dimethylthiazol-2-yl)-2,5-diphenyltetrazolium bromide (MTT) (Sigma-Aldrich, St. Louis, MI, USA) assay [47].

### 4.4. Cytokines Release

Quantities of IL-1α, IL-1β, IL4, MCP-1, IL10, IL12p40, IL12p70, IL6, IL13, GM-CSF, G-CSF, EOTOXIN, IL-17, IL19α, TNF-α, IFNγ, RANTES, MIP-1α, MIP-1β and KC were determined by Magpix powered by Luminex XMAP technology. The assays were conducted in plates with 96 wells, with the aid of high-sensitivity RCYTOMAG 80K panels (Millipore Corporation, Billerica, MA, USA), according to the manufacturer’s instructions. The amount of each analyte was expressed in pg/µL. Samples were assessed in triplicate and the mean values found were utilized to determine the concentrations of each marker [11].

### 4.5. Analysis of Gene Expression by Real-Time PCR

Gene expression was assessed by reverse transcription followed by real-time PCR. Total RNA was obtained from macrophages activated with LPS and treated with V55 and vehicle-control plus LPS, using a kit for RNA extraction (Qiagen, Hilden, Germany). First strand synthesis was achieved with 1 μg of RNA using an RT2 First Strand Kit (Qiagen). PCR was conducted using the mouse Signal Transduction Pathway (PAMM 014CZ) array; the mouse phosphoinositide 3-kinase-protein kinase B array (PI3K-AKT) signaling pathway (PAMM-058CZ); the nitric oxide signaling pathway array (PAMM-062CZ) and the mouse common cytokines array (PAMM-021CZ) (Qiagen). In total, three hundred and sixty genes were evaluated. Differences in the expression of the target genes were calculated in relation to the mean cycle threshold (CT) numbers of five distinct calibrator genes (*Gusb*, *Hprt*, *Hsp90ab1*, *Gapdh* and *Actb*) by the ΔΔCT method [26].

### 4.6. Statistical Analysis

Data from cell viability and NO release among the groups were analyzed utilizing ANOVA followed by Tukey–Kramer, using the Biostat Software. For cytokines release, a Mann–Whitney test was conducted. For the PCR array, the student’s t-test was performed using CT values of (3*S*)-vestitol and control (DMSO) treated groups (*p* < 0.05), using the SABiosciences Technical Core website (SABiosciences/Qiagen Corp., Frederick, MD, USA).

## 5. Conclusions

(3*S*)-vestitol 0.55 µM showed outstanding anti-inflammatory properties based on the data from the present manuscript. Molecular mechanisms of (3*S*)-vestitol’s anti-inflammatory effect comprise cytokines and NF-κB pathway inhibition such as inhibition of IL-1β, IL-1α, G-CSF, IL-10 and GM-CSF levels and down-regulation of the expression of *Icam-1*, *Wnt5a* and *Mmp7* (related to inflammatory response and periodontal tissue destruction). Moreover, (3*S*)-vestitol up-regulated the expression of *Socs3* and *Dab2* genes (inhibitors of cytokine signaling and the NF-κB pathway), *Apoe* (related to atherosclerosis control), *Igf1* (encoder a protein with analogous effects to insulin) and *Fgf10* (fibroblasts growth factor). Besides, (3*S*)-vestitol is a promising option for future in vivo investigations of the treatment/prevention of chronic inflammatory disorders such as periodontal disease and atherosclerosis.

## Data Availability

The data presented in this study are available on request from the corresponding author.
